# Supplementary material for: Scaling up DNA digital data storage by efficiently predicting DNA hybridisation using deep learning
Source: Sci Rep. 2021 Oct 15;11:20517. doi: 10.1038/s41598-021-97238-y (PMC8519920; doi:10.1038/s41598-021-97238-y)
Supplement: Supplementary file 1 — Supplementary Information. [file 41598_2021_97238_MOESM1_ESM.pdf]

Supplementary information

1 Alignment scores

Notice that there are discrepancies between the parasail alignments below and the structures reported by NUPACK in Figure 3a and Figure 3b.

Score 130 - **Yield** 1.0:

```
GAATACTGTCAGTGAGAGGATCTGCC
||||||||||||||||||||||||||
GAATACTGTCAGTGAGAGGATCTGCC
```

Score 77 - **Yield** 0.12:

```
TTGTCATACGCTGTAAGAG
|||||||.|||||||||.|
TTGTCATCCGCTGTAAGCG
```

Score 31 - **Yield** 0.20:

```
----C-TGCGGCGCCGTTTGCATGCTCTCG
    | ..|||||||    ||| |
AGAGCAAACGGCGCC----GCA-G------
```

Score 86 - **Yield** 0.82:

```
AAATCAGGTA---TGCGGTAAG
||||||||||   |||||||||
AAATCAGGTACGTTGCGGTAAG
```

2 Hyperparameters for LDA, QDA, RF and NN

The hyperparameter optimisation for the baseline machine learning algorithms is performed using sklearn [33], more specifically with the `GridSearchCV` class configured to use our validation split (possible using `PredefinedSplit`). The monitored metric is the Matthews Correlation Coefficient, as advised in [28], [29]. The chosen search space is inspired by the default hyperparameter settings and is meant to provide reasonable coverage, keeping in mind a trade-off between exhaustiveness and time.

LDA

For LDA, the number of hyperparameters is small. We use a parameter grid with two entries:

```
{solver: [lsqr, eigen], shrinkage: [auto, *np.linspace(0.0, 1.0, num=11)]},
{solver: [svd]}
```

The found (and used) hyperparameters are: `solver: eigen, shrinkage: 0`.

Random forests

For random forests, the search space included:

```
{n_estimators: [30, 100, 200], max_depth: [None, 10, 30, 100],
max_features = [None, auto, sqrt], min_samples_split = [2, 5],
min_samples_leaf = [1, 2, 4], bootstrap = [True, False]}
```

The returned hyperparameters were `bootstrap: True, max_depth: 10, max_features: sqrt min_samples_leaf: 1, min_samples_split: 2, n_estimators: 100`. However, training with default parameters resulted in better performance on the test set. Thus, the reported results make use of the default hyperparameters.

Neural networks

In the case of neural networks we tuned hyperparameters using Optuna [34], an open-source optimisation framework that includes state-of-the-art algorithms. In particular, Optuna also includes a pruning capability to stop training unpromising trials. The hyperparameters included in the search are: the number of hidden dense layers `n_layers` $\in \{1, 2, 3, 4, 5\}$, a single dropout value to be applied after every

hidden layer `dropout` $\in [0.2, 0.5]$, the number of neurons per hidden layer `n_units` $\in \{4, 5, ..., 128\}$, the batch size `batch` $\in \{32, 64, 128, 256, 512\}$ and the learning rate `lr` $\in [0.0001, 0.1]$ (log domain). The neural optimisation algorithm is Adam [35].

Optuna was run with the `HyperbandPruner` for 50 trials with the goal of minimising the validation loss (binary cross-entropy as it is a classification task). The returned (and used) best hyperparameters were: `n_layers: 4, dropout: 0.3296, n_units_l0: 117, n_units_l1: 18, n_units_l2: 7, n_units_l3: 19, batch: 1024, lr: 0.0002` (rounded to 4 decimal places).

3 Classification metrics for LDA, QDA, RF and NN

The following table provides the basis for our discussion on the classification performance of the baseline ML models.

Supplementary Table 1: Precision, recall and F_1 corresponding to Figure 4c.

	**LDA**		**QDA**		**RF**		**NN**	
**Metric**	**Low**	**High**	**Low**	**High**	**Low**	**High**	**Low**	**High**
**Precision**	0.996	0.881	0.975	0.901	0.966	0.927	0.971	0.914
**Recall**	0.827	0.998	0.860	0.983	0.901	0.975	0.881	0.980
$\mathbf{F_1}$	0.904	0.936	0.914	0.940	0.932	0.950	0.924	0.946

4 Classification metrics for CNN, RNN and RoBERTa

Similarly to the above, we report the classification metrics for the deep learning models after applying the 0.2 threshold on the predicted yields (binarisation).

Supplementary Table 2: Precision, recall and F_1 corresponding to Figure 4d.

	**RNN**		**CNN**		$\mathbf{CNN_{Lite}}$		**RoBERTa**	
**Metric**	**Low**	**High**	**Low**	**High**	**Low**	**High**	**Low**	**High**
**Precision**	0.984	0.960	0.989	0.941	0.990	0.931	0.985	0.898
**Recall**	0.948	0.988	0.920	0.992	0.906	0.993	0.855	0.990
$\mathbf{F_1}$	0.965	0.974	0.953	0.966	0.946	0.961	0.915	0.942

5 Hyperparameters for CNN, RNN and RoBERTa

As the three architectures have innate differences, our approach to designing each model is different.

CNN

For our CNN model, we follow a top-down approach based on the properties of our inputs (pairs of DNA sequences represented as grids). At a high level, the network layers can be grouped in convolutional blocks, where each block has a convolutional layer, an activation (ReLU) layer and a batch normalisation layer (in this order), with dropout layers interspersed according to Supplementary Table 3. The model is trained with the Adam optimiser and a learning rate of 0.0001, a batch size of 256 (maximum that would fit in 8GB of GPU memory) with the Minimum Squared Error (MSE) loss and early stopping set to a patience of 3 epochs and no maximum number of epochs.

We also performed hyperparameters search for the CNN architecture. The search space was defined by: number and size of filters `conv2d_size` $\in \{9, 10, 11, 12, 13\}$, `conv1d_size1` $\in \{7, 8, 9, 10, 11\}$, `conv1d_size2`, `conv1d_size3` $\in \{3, 4, 5, 6, 7\}$, `conv1d_size4` $\in \{1, 2, 3\}$, `conv2d_filter`, `conv1d_filter1`, `conv1d_filter2` $\in \{256, ..., 768\}$, `conv1d_filter3` $\in \{128, ..., 384\}$, `conv1d_filter4` $\in \{32, ..., 128\}$, the batch size `batch_size` $\in \{256, 512, 1024, 2048, 4096, 8096\}$ and the learning rate `lr` $\in [0.0001, 0.1]$ (log domain). Dropout can be applied after each convolutional layer, with a choice of probability in $[0, 0.5]$ and after the linear layers with probability in $[0.1, 0.5]$. The choice of size for the linear layers is based on the value selected for `conv1d_filter4`. The values found by the hyperparameter search are available in the repository. However, they were not used as the classification performance on the test set using the reported hyperparameters was slightly worse than the architecture described in Supplementary Table 3.

Supplementary Table 3: Overview of the CNN architecture.

Layer	In channels	Out channels	Filter or dropout
2D Convolution	2	512	4×9
Dropout	*	*	0.2
1D Convolution	512	512	9
1D Convolution	512	128	3
Dropout	*	*	0.2
1D Convolution	128	128	3
1D Convolution	128	64	1
Fully connected	384	256	*
Fully connected	256	128	*
Dropout	*	*	0.2
Fully connected	128	1	*

Supplementary Table 4: Overview of the CNN_{Lite} architecture.

Layer	In channels	Out channels	Filter or dropout
2D Convolution	2	256	4×9
Dropout	*	*	0.2
1D Convolution	256	128	9
1D Convolution	128	64	3
Fully connected	512	256	*
Dropout	*	*	0.2
Fully connected	256	1	*

RNN

The RNN architecture includes an embedding layer, a configurable multi-layer bi-directional LSTM block, a dropout layer and the fully-connected regression layer (in this order). The parameters of the LSTM-based model are not as intuitive as their CNN counterpart and we address this issue by performing hyperparameter optimisation using Optuna. The search space includes: the embedding dimension `emb_dim` $\in \{16, 32, 64\}$, the number of LSTM layers `n_layers` $\in \{1, 2, 3, 4\}$, the LSTM dropout `lstm_dropout` $\in [0.1, 0.5]$, the number of features in the hidden state `hidden` $\in \{32, 64, 100\}$, the dropout preceding the regression layer `lin_dropout` $\in [0.1, 0.5]$, the batch size `batch` $\in \{64, 128, 256\}$ and the learning rate `lr` $\in [0.0001, 0.1]$ (log domain). As before, the model is trained with the MSE loss and early stopping set to a patience of 3 epochs and no maximum number of epochs.

The returned (and used) RNN hyperparameters are `emb_dim: 32, lstm_dropout: 0.2412 n_layers: 3, hidden: 64, lin_dropout: 0.2166, batch: 128, lr: 0.006` (rounded to 4 decimal places).

RoBERTa

Transformer models require substantially more computing power to train end-to-end (including both the pre-training and the fine-tuning stages). As such, it is not feasible to perform hyperparameter optimisation for RoBERTa. However, we base our configuration on known RoBERTa architectures such as `roberta-base` and on the intuition that modelling DNA sequence hybridisation is an easier task than natural language processing.

As described in the main text, the Transformer model is first pre-trained on a Masked Language Model (MLM) task after the inputs have been tokenised with `RobertaTokenizerFast` (the provided tokenisation class). The pre-training phase uses the following hyperparameters: vocabulary size `vocab_size` = 5000, dimension of the encoder and pooler layers `hidden_size` = 256, number of encoder hidden layers `num_hidden_layers` = 6, number of attention heads for each attention layer of the encoder `num_attention_heads` = 8, dimensionality of the intermediate layer `intermediate_size` = 1024, the maximum sequence length that the model might ever use `max_position_embeddings` = 128, vocabulary setting `type_vocab_size` = 1, dropout probability for all fully connected layers in the embeddings `hidden_dropout_prob` = 0.3 (also in the encoder and pooler), dropout ratio of attention probabilities `attention_probs_dropout_prob` = 0.3, Masked Language Model masking probability `mlm_probability` = 0.15, number of warm-up steps `warmup_steps` = 500, number of per-device batch size `per_device_train_batch_size` = 256 and the total number of pre-training epochs

`num_train_epochs` = 6.

The other parameters are left to the default values. Our higher than default dropout values are used to help combat potential overfitting in the case where the MLM training task is too easy on our sequences. We find that around 4-6 epochs are enough for the model to converge on the pre-training phase.

For the fine-tuning phase, we load the entire pre-trained RoBERTa model with trainable weights and append a dropout layer with probability 0.1 and a dense layer for regression of the same size as the encoder layers (256). The input preprocessing employs the pair encoding capabilities of the tokeniser (initially designed for sentence pair tasks) and is very similar to the RNN encoding with special characters from Figure 4b. The resulting network is trained with the MSE loss, AdamW optimiser with a learning rate of 0.00002 and weight decay set to 0.01, with early stopping set to a patience of 3 epochs and no maximum number of epochs.

6 Background on Transformers

The term Transformer usually translates to a multi-layer architecture built from composable, structurally-identical Transformer *blocks*, the fundamental units. The input to such a block is a d-dimensional vector, the additive composition of the input embedding vector itself with a separately-computed positional embedding vector. An important characteristic of the Transformer block is the self-attention mechanism, which computes an $N \times N$ attention matrix, with N the sequence length. This procedure allows tokens to attend to other, possibly distant tokens which are deemed relevant (i.e., high attention scores). Having k-head attention corresponds to k independent and concurrent attention computations that can be concatenated or summed. As noted in [21], Transformer blocks can be employed for different purposes. The three classic cases are *encoder-only*, *decoder-only* and *encoder-decoder*, where each comes with specific implementation decisions and design choices. Naturally, the above computation is quadratic, and many recent variations have been proposed to combat this lack of efficiency [21].

The original pre-training procedure includes two training objectives: Masked Language Model (MLM) and Next Sentence Prediction (NSP). MLM randomly replaces a per cent of input tokens with a special *mask* token and learns to predict the missing character. NSP is a classification problem where the objective is to predict if two sentences follow each other in the input text.

A possible extension is the *Vision Transformer* [36], a Transformer architecture designed to work on images, offering competitive performance with deep CNNs while requiring less resources. We do not investigate Vision Transformers in this work as the inference times will likely lag behind our relatively shallow CNNs, while the regression performance is also unlikely to be stellar considering our results with the sequence-based Transformer.

7 Clustering with MMseqs2

A small sample of the 39,432,713 output pairs is reproduced in Supplementary Table 5. Notice that for each ID in the first column, there are multiple sequences which are deemed similar by MMSeqs2. For example, the sequence denoted by ID `seq43840` has 8 other similar sequences associated with it. This is however just a subset of all the sequences found to be similar with `seq43840` and reproduced here for illustration purposes.

8 Experimental platform

The various experiments in this study are performed on a number of different platforms to accommodate their unique requirements. Hyperparameter optimisation, general non-GPU (Graphics Processing

Supplementary Table 5: Small sample of the MMseqs2 output.

**ID 1**	**ID 2**	**Sequence 1**	**Sequence 2**
seq43840	seq49789	GCGCCACCGCGTATATTAGG	AAGCTTAATACACGCGGTGC
seq43840	seq81414	GCGCCACCGCGTATATTAGG	ATACGCGGTGGATGCGTAGC
seq43840	seq92992	GCGCCACCGCGTATATTAGG	CATACGCGGCGGCGTCATAA
seq43840	seq11505	GCGCCACCGCGTATATTAGG	GTTCTAATCTACGCGGAGTC
seq43840	seq82545	GCGCCACCGCGTATATTAGG	GCACTTCATATATGCGGTGG
seq43840	seq19444	GCGCCACCGCGTATATTAGG	ATACGCCGTGGCGGCCGGCA
seq43840	seq15232	GCGCCACCGCGTATATTAGG	ATAATACGCGGTGAGTATAA
seq43840	seq25057	GCGCCACCGCGTATATTAGG	TTCTAATATACGACCTGACA
seq43968	seq59355	AAATAGCCTTTACTATGTCC	CTCGCAGTAAAGGCACCACC
...	...	...	...

Unit) intensive tasks and training of the simpler neural models (feed-forward neural networks) were performed on a portable computer equipped with a Core i9-8950HK processor, 32GB of DDR4 RAM, a PCI Express 3.0 solid-state drive (SSD) and an NVIDIA RTX 2070 external GPU connected through Thunderbolt 3, with 8GB VRAM.

RoBERTa pre-training was performed on the Google Cloud Platform with 8 TPUv3 (third generation) cores and fine-tuned on a system equipped with 8 vCPUs, 61GB RAM, an SSD and an NVIDIA Tesla V100 with 16GB VRAM. Some compute-intensive tasks such as NUPACK or MMseqs2 were either run on our portable platform or an Azure virtual machine (VM) with 64 vCPUs and a premium SSD, as indicated in the main text. Due to the different configurations, we do not report *training* times.

Experiments involving the measurement of time were performed on three platforms. NUPACK code, which runs only on the Central Processing Unit (CPU) was timed on an Azure VM equipped with the hardware described above. The rest of the timing experiments were performed, by design, on consumer hardware. The two chosen consumer platforms are (1) a computer equipped with an AMD Ryzen 5950X CPU, 32GB of DDR4 RAM, a PCI Express 4.0 SSD and an NVIDIA RTX 3090 GPU with 24GB of VRAM and (2) Google Colaboratory with 8 TPUv2 cores (second generation).

9 Software

All code is written in the Python programming language and has been confirmed as working under revisions 3.8.5 and 3.8.6. The required machine learning libraries include scikit-learn 0.24, PyTorch 1.7.1 and PyTorch Lightning 1.1.4, which were the latest versions available as of January 2021. The main development operating system on the portable platform and the RTX 3090 GPU platform is Windows 10 Insider Preview Build 21286 (available on the Dev Channel), while the other platforms use various recent distributions of Linux.

NUPACK, parasail and MMseqs2 computations were performed on the same portable computing platform as above, using their respective most recent versions as of May 2019. This translates to NUPACK

version 3.2.2 and parasail versions 2.4.1 and 2.4.2. Our original CNN implementation (not presented in this paper) employed the most up-to-date TensorFlow 1 and Keras versions as of May 2019.

10 Measuring inference time

The timing code for GPUs is based on the following snippet:

```
start = torch.cuda.Event(enable_timing=True)
end = torch.cuda.Event(enable_timing=True)

with torch.no_grad():
    start.record()
    trainer.test(model, test_dataloader)
    end.record()
    torch.cuda.synchronize()
    elapsed_time = start.elapsed_time(end)
```

The code can be trivially extended to perform multiple repetitions of the same code block in order to report the average execution time and standard deviation.

Omitting the non-timing code, the timing code structure for TPUs is as follows:

```
class Module(pl.LightningModule):
    def __init__(self, ...):
        super(Module, self).__init__()
        self.forward_flag = 0

    def forward(self, x):
        if not self.forward_flag:
            self.forward_flag = 1
            self.start = time.time()

        x = ...

    def on_test_epoch_end(self):
        end = time.time()
        elapsed_time = end - self.start
```

This introduced probe effect will alter the execution time, but we assume the impact is negligible. Importantly, the above code is run on all of the 8 available TPU cores, meaning that each core reports its own time. As the cores operate concurrently, we take the mean of the 8 elapsed times and assign the result to the corresponding trial.

It is also important to note that raw GPU performance roughly doubles with each generation, a trend also observed by Tensor Processing Units. Thus, the deep learning models we have developed will continue to improve in terms of inference time without any additional effort. A further optimisation would be the use of 16-bit precision. For a minor accuracy penalty, 16-bit implementations can both reduce video memory usage and improve execution times by several factors in the range of $\times 1 - 5$.

Furthermore, at the time of writing, the latest NVIDIA GPU architecture, Ampere, is still not fully exploited under CUDA Toolkit 11.0, the official version supported by PyTorch 1.7.1. For this reason, we compiled from source a nightly version of PyTorch 1.8.0 with CUDA 11.1 and this setup was used for all GPU timing experiments. However, it is likely that further optimisations will be possible on the Ampere architecture.

11 Detailed performance metrics on other temperatures

Supplementary Table 6 presents the MSE, MCC and AUROC for the five evaluated temperatures and Supplementary Table 7 lists per-class precision, recall and F_1 scores.

Supplementary Table 6: Summary of the evaluation metrics for the deep learning models at the selected temperatures.

**Model**	**Metric**	**37.0C**	**42.0C**	**47.0C**	**52.0C**	**62.0C**
RNN	MSE	808.126	655.260	433.125	190.772	333.637
	MCC	0.862	0.872	0.888	0.916	0.833
	AUROC	0.946	0.949	0.954	0.963	0.911
CNN	MSE	719.616	576.134	374.768	170.865	398.881
	MCC	0.884	0.893	0.908	0.929	0.811
	AUROC	0.955	0.958	0.962	0.967	0.899
CNN_{Lite}	MSE	712.598	574.295	381.676	188.943	418.637
	MCC	0.893	0.901	0.914	0.929	0.801
	AUROC	0.958	0.961	0.964	0.966	0.892
RoBERTa	MSE	546.058	452.876	336.600	249.726	674.329
	MCC	0.925	0.930	0.930	0.914	0.762
	AUROC	0.971	0.971	0.968	0.954	0.870

Supplementary Table 7: Precision, recall and F_1 scores for the **Low** and **High** classes resulting from the deep learning models at the selected temperatures.

CNNLite

Metric	37.0C		42.0C		47.0C		52.0C		62.0C	
**Precision**	0.872	0.998	0.885	0.996	0.908	0.992	0.947	0.979	0.999	0.817
**Recall**	0.996	0.921	0.993	0.929	0.987	0.942	0.967	0.965	0.785	0.999
$\mathbf{F_1}$	0.930	0.958	0.936	0.961	0.946	0.966	0.957	0.972	0.879	0.899

CNN

Metric	37.0C		42.0C		47.0C		52.0C		62.0C	
**Precision**	0.860	0.999	0.873	0.997	0.897	0.995	0.940	0.984	0.999	0.826
**Recall**	0.998	0.912	0.996	0.920	0.991	0.934	0.975	0.960	0.798	0.999
$\mathbf{F_1}$	0.924	0.953	0.931	0.957	0.942	0.963	0.957	0.971	0.887	0.905

RNN

Metric	37.0C		42.0C		47.0C		52.0C		62.0C	
**Precision**	0.835	0.999	0.848	0.998	0.872	0.996	0.918	0.988	0.999	0.844
**Recall**	0.998	0.893	0.997	0.901	0.993	0.915	0.982	0.944	0.822	1.000
$\mathbf{F_1}$	0.909	0.943	0.917	0.947	0.929	0.954	0.949	0.965	0.902	0.915

RoBERTa

Metric	37.0C		42.0C		47.0C		52.0C		62.0C	
**Precision**	0.915	0.995	0.926	0.992	0.942	0.983	0.962	0.957	0.996	0.788
**Recall**	0.991	0.950	0.986	0.956	0.971	0.965	0.932	0.976	0.743	0.997
$\mathbf{F_1}$	0.951	0.972	0.955	0.974	0.956	0.974	0.947	0.967	0.851	0.881

12 Ablation study

To study the effectiveness of the different extracted features, we perform an ablation study examining the classification performance of various configurations of interest. The results are presented in Supplementary Table 8, Supplementary Table 9, Supplementary Table 10, Supplementary Table 11. There are less entries for NN due to extended training time.

Supplementary Table 8: Ablation study summary for LDA.

**Aln.**	**GC**	**S.C.**	**P.C.**	**S. MFE**	**MCC**	**AUROC**
✓					0.840	0.906
✓	✓				0.846	0.909
✓	✓	✓			0.846	0.909
✓	✓		✓		0.846	0.909
✓	✓			✓	0.850	0.912
✓	✓		✓	✓	0.850	0.912
✓	✓	✓		✓	0.850	0.912
✓	✓	✓	✓	✓	**0.851**	**0.912**
		✓			0.000	0.500
			✓		0.000	0.500
				✓	0.000	0.500
	✓	✓	✓	✓	0.033	0.502

Supplementary Table 9: Ablation study summary for QDA.

**Aln.**	**GC**	**S.C.**	**P.C.**	**S. MFE**	**MCC**	**AUROC**
✓					0.845	0.910
✓	✓				0.853	0.916
✓	✓	✓			0.852	0.918
✓	✓		✓		0.852	0.918
✓	✓			✓	0.858	0.920
✓	✓		✓	✓	0.859	0.922
✓	✓	✓		✓	0.859	0.922
✓	✓	✓	✓	✓	**0.859**	**0.922**
		✓			0.023	0.504
			✓		0.023	0.504
				✓	0.003	0.501
	✓	✓	✓	✓	0.096	0.548

The table headers refer to the concepts introduced in *Designing a diverse hybridisation dataset* (Aln., Alignment, GC, GC content, S.C., Single Concentration, P.C. Pair Concentration, S. MFE, Single MFE, MCC, Matthews Correlation Coefficient, AUROC, Area Under the Receiver Operating Characteristics).

Supplementary Table 10: Ablation study summary for RF.

**Aln.**	**GC**	**S.C.**	**P.C.**	**S. MFE**	**MCC**	**AUROC**
✓					0.848	0.913
✓	✓				0.884	0.937
✓	✓	✓			0.875	0.934
✓	✓		✓		0.875	0.933
✓	✓			✓	**0.884**	**0.938**
✓	✓		✓	✓	0.879	0.935
✓	✓	✓		✓	0.880	0.936
✓	✓	✓	✓	✓	0.879	0.935
		✓			0.141	0.547
			✓		0.151	0.553
				✓	0.190	0.565
	✓	✓	✓	✓	0.559	0.780

Supplementary Table 11: Ablation study summary for NN.

**Aln.**	**GC**	**S.C.**	**P.C.**	**S. MFE**	**MCC**	**AUROC**
✓	✓				0.867	0.931
✓	✓			✓	0.872	0.932
✓	✓	✓	✓	✓	**0.873**	**0.930**

It is clear that alignment scores alone are a good predictor of hybridisation yield. A modest, but visible increase in classification performance is further provided by the quick-to-compute GC content. Depending on the machine learning model, the benefit of adding thermodynamic information is more or less subtle. In Supplementary Table 8, Supplementary Table 9, Supplementary Table 10, Supplementary Table 11 the best scores are highlighted in bold: first by MCC; in case of equality also by AUROC; in case of equality of AUROC the most complete model (most features) is highlighted. For LDA, QDA and NN the best performing models use all features. For RF, the Aln-GC model has nearly identical performance to the Aln-GC-MFE model.

However, the differences are statistically significant: for LDA, between the complete model and the Aln-GC variant ($P = .034$, permutation test with 5000 iterations, thus significant at the 95% confidence interval); for QDA, between the complete model and the Aln-GC variant ($P < .001$, permutation test with 5000 iterations, thus significant at the 99% confidence interval); for RF, between the Aln-GC-MFE

model and the Aln-GC variant ($P < .001$, permutation test with 5000 iterations, thus significant at the 99% confidence interval) and for NN between the complete model and the Aln-GC variant ($P < .001$, permutation test with 5000 iterations, thus significant at the 99% confidence interval). The null hypothesis that the two groups come from the same distribution is rejected in all cases at the mentioned confidence interval. This two-sided permutation test is provided by the `permutation_test` function of the mlxtend [37] Python library. The statistical difference in otherwise close scores can be explained by trade-offs made by the model: some models might favour reducing false positives over false negatives and vice-versa.

13 Inference times for different dataset sizes

The inference time on second generation TPUs was measured using the methodology described in Supplementary Information 10, for subsets of size: 250,000, 500,000, 1,000,000, 2,000,000 and 2,556,976 (full dataset). These datasets can be entirely loaded in memory, hence a PyTorch `TensorDataset` (without shuffling) was used to hold the data, initially loaded with NumPy [38]. We further measured inference time on a dataset of size 5,113,952 (double the full dataset length), by concatenating two `TensorDataset` objects holding the 2.5 million data points using PyTorch's `ConcatDataset`. The batch size was set to 8,192 as it enables better scalability on the larger datasets.

Supplementary Figure 1 illustrates the measured inference time as well as the extrapolated values based on two reference points: 500,000 and 1,000,000, called "500K projection" and respectively "1M projection", for the three most efficient algorithms: RNN, CNN and CNN_{Lite}.

All evaluated models see a spike in inference time for the 1 million dataset, however this trend improves for the RNN, whose times for the > 1 million datasets stay below the 1M projected line (only $\times 1.84$ increase from 2.5M to 5M). The CNN and CNN_{Lite} follow the 1M projected line closely until around 2.5M in dataset size. Afterwards, the difference compared to the 1M projected line increases; however the actual measured time increases by a factor of about $\times 2.27$ for CNN_{Lite} and $\times 2.30$ for the CNN when transitioning from the 2.5M dataset to the 5M dataset. Thus, both the CNN and CNN_{Lite} models are close the the ideal $\times 2$ increase in inference time as the dataset size doubles; in addition, the times are better than the RNN, which scales better than linearly relative to its own performance.

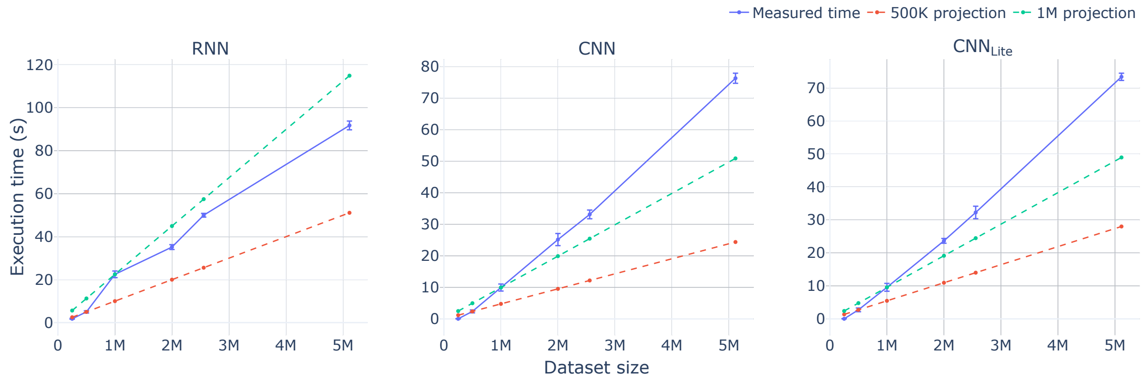

Supplementary Figure 1: Scatter plots of the inference times for the three most efficient models: RNN, CNN and CNN_{Lite} as the dataset size increases, together with projections based on the times for the 500K and 1M datasets. The batch size is set to 8,192. In the graphs, M is used for million(s).

14 Similarity between yields at different temperatures

An intuition for the similarity between the ground truth yields as computed by NUPACK at different temperatures can be attained by pairwise scoring the values using a criterion such as mean absolute error (MAE) or mean squared error (MSE). For this comparison, all yields are in the range $[0, 1]$. Supplementary Figure 2 captures these metrics and indicates that the yields at 62°C are the most different, even when compared to their closest neighbour (57°C). On the other hand, yields in the range 37°C to 52 °C are relatively close to each other (in all cases MAE lower than 0.1, MSE lower than 0.04).

The choice of 57°C for training and evaluation is motivated by being close to the standard melting and/or annealing temperatures of PCR primers (more details in the main text) and not being too far away from the behaviours at 62°C and < 57°C as illustrated in Supplementary Figure 2.

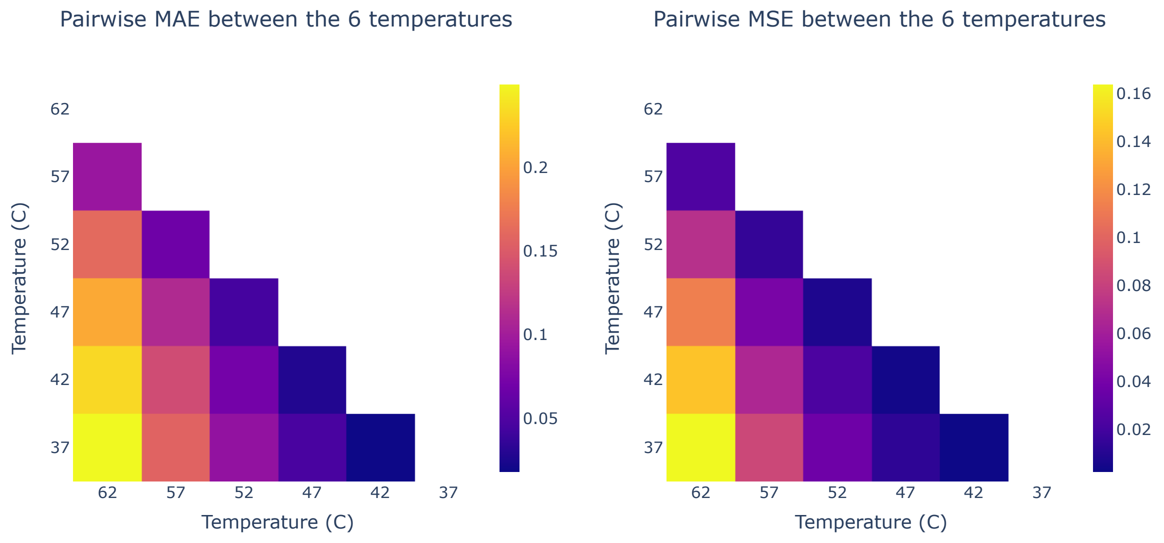

Supplementary Figure 2: The ground truth yields at the six different temperatures are compared in a pairwise manner to assess their relatedness, using the MAE and MSE as the scoring functions.
